# Normative isokinetic knee strength values and prediction models in non-athletic Chinese adults

**DOI:** 10.3389/fbioe.2025.1573267

**Published:** 2025-05-21

**Authors:** Kun Liu, Lulu Yin, Ye Zhang, Gongliang Liu, Ping Fang, Yanhong Ma, Lihua Huang

**Affiliations:** ^1^ Department of Rehabilitation Medicine, Shanghai Sixth People’s Hospital, Shanghai, China; ^2^ Key Laboratory of Exercise and Health Sciences (Shanghai University of Sport), Ministry of Education, Shanghai, China

**Keywords:** isokinetic strength, knee joint, normal reference value, machine learning, prediction models

## Abstract

**Background:**

Establishing normative isokinetic muscle strength values and prediction models for knee joints in non-athletic healthy Chinese adults aids in clinical assessments, diagnosis, and rehabilitation. However, there has been limited research on these normal reference values, particularly involving large sample cohorts. Therefore, this study aimed to develop normative reference values and predictive models for knee joint isokinetic muscle strength across different age groups and genders in non-athletic healthy Chinese adults.

**Methods:**

A total of 2,208 participants aged between 20–70 years old were included in the study. The participants were divided into two groups by gender and further divided into five age groups: 20–29, 30–39, 40–49, 50–59 and 60–69 years old, resulting in a total of 10 groups. Key parameters, including peak torque of knee flexors and extensors, relative peak torque, peak torque ratio of flexors to extensors, peak torque angle and work, were collected using an isokinetic dynamometer at angular velocities of 60°/s and 180°/s. Two-way analysis of variance was utilised to analyse the characteristics and differences of these parameters amongst different age groups and genders. Pearson correlation coefficients were calculated to examine the relationships between these parameters and gender, age, height, weight and body mass index. Predictive models were developed using linear regression and various machine learning techniques.

**Results:**

Males exhibited significantly higher knee isokinetic strength values than females across all age groups, with knee extensor strength 20.47%–38.01% higher and knee flexor strength 22.91%–43.42% higher at both 60°/s and 180°/s. Muscle strength showed a moderate negative correlation with age, indicating a decline with increasing age. Extension strength values were greater than flexion, and measurements at 180°/s were lower compared to 60°/s. The multilayer perceptron regressor demonstrated the highest predictive capability among the models tested.

**Conclusion:**

This study provides comprehensive normative reference values and predictive models for knee joint isokinetic muscle strength in non-athletic healthy Chinese adults. The results highlight significant gender and age differences, offering valuable data for clinical assessments and personalized rehabilitation strategies to improve knee joint health and overall quality of life.

## Introduction

Muscle strength is a cornerstone of health and physical fitness, capable of preventing chronic diseases and regarded as the most significant predictor of function and physical performance (Volaklis et al., 2015; Weigent et al., 1998; Garber et al., 2011). Meanwhile, muscle weakness is considered a risk factor for high mortality rates in the elderly (Baumgartner et al., 1999; Jones et al., 2009; Doherty, 2001). In daily life and clinical rehabilitation settings, the demand for assessing muscle strength is high. Such assessment not only aids in diagnosing the severity of muscle weakness but also facilitates monitoring the effectiveness of exercise prescriptions and intervention programs (Jones, 2000).

Isokinetic dynamometry, a computerised apparatus for muscle strength assessment (Li et al., 2006), is regarded as the gold standard for evaluating muscular strength in the clinical and research settings (Dvir, 1996; van Dyk et al., 2016). This technique measures specific parameters, such as peak torque (PT) and work, at constant velocities, providing reliable and objective data. These measurements are crucial for identifying strength deficits and evaluating the effectiveness of interventions on muscle strength (van Dyk et al., 2016; Perrin et al., 1987; Coudeyre et al., 2016). Over the past few decades, studies in developed countries have established normative reference values and prediction equations for muscle strength in various populations (Schindler et al., 2023; Benfica et al., 2018), including athletes (Zvijac et al., 2014; Risberg et al., 2018; Hannon et al., 2022), children (Holm et al., 2008; McKay et al., 2017), older adults (Pereira et al., 2019a; Bohannon, 2017) and general adult populations. (McKay et al., 2017; Meldrum et al., 2007; Harbo et al., 2012). These references serve as essential benchmarks for identifying strength deficits, setting rehabilitation targets, and evaluating intervention outcomes (Meldrum et al., 2007; Mathiowetz et al., 1985; Peolsson et al., 2001).

Despite these advancements, most studies has been concentrated in developed countries in the Northern Hemisphere. While in developing nations, only a small number, such as Brazil (Pereira et al., 2019b), India, (Sucharita et al., 2023), Iran (Rezaei et al., 2014) and Saudi Arabia (Alangari and Al-Hazzaa, 2004), have reported isokinetic muscle strength reference values or prediction equations for their populations. Increasing dietary protein intake has been reported to significantly improve thigh muscle quality and strength (Pasiakos and Howard, 2021; Hartono et al., 2022). Despite a substantial increase in the *per capita* consumption of animal-based foods, such as meat, eggs and dairy over recent decades, the dietary structure and protein intake levels amongst residents of countries at different stages of development show significant disparities (Miller et al., 2022). This situation results in variations in body mass index, muscle mass index, height, weight and other characteristics amongst racial and demographic groups in different countries, which may influence the results of muscle strength tests (Baker et al., 2013). The aforementioned situation underscores the necessity of separately measuring and establishing muscle strength reference values for populations with specific demographic characteristics (Zengin et al., 2015).

To the best of our knowledge, a limited number of research has been carried out on normal reference values for isokinetic knee joint muscle strength amongst non-athletic healthy adults in China, particularly in large sample cohorts. Additionally, the predominantly East Asian population may possess different strength characteristics compared with populations in other geographical regions. Only a few studies have reported the isokinetic muscle strength values of the knee joints in the Japanese population; however, due to the small sample size and the outdated data, it is difficult to provide precise references for clinical applications in the current Chinese population (Kanehisa et al., 1994; Akima et al., 2001). Given the Chinese population’s vast size, diverse genetic backgrounds, lifestyles and environmental factors, Therefore, indigenous normative reference values and prediction models must be established for isokinetic knee joint muscle strength in China.

Nowadays, machine learning techniques have been utilized in risk analysis quantification and identification in the biomechanical field (Nolte et al., 2025; Bodkin et al., 2022; Lu et al., 2024), and they have become increasingly prevalent in predicting disease occurrence and facilitating rehabilitation applications due to the advancements in science and technology (Wei et al., 2024; Espinoza Bernal et al., 2021; Powers et al., 2023; Uddin et al., 2019).Unlike traditional linear regression models (Pereira et al., 2019b; Gülü and Akalan, 2021; Zhang Y. et al., 2023), machine learning can capture complex, non-linear relationships between key variables that linear models may not fully address (Wei et al., 2024). In this study, machine learning techniques will be applied to analyze large datasets of isokinetic knee joint muscle strength and related factors, such as age, gender, and other demographic characteristics. This study aims to fill the research gap in normative reference values for isokinetic knee joint muscle strength in non-athletic healthy adults in China. Additionally, this study seeks to compare various machine learning models, including linear regression equations, to identify the optimal predictive model for isokinetic muscle strength. This endeavour will provide crucial data support for improving knee joint health, reducing disease risks and enhancing the overall quality of life.

## Methods

### Participants

This study is a cross-sectional investigation. Approximately 2,500 adults aged 20–69 years old were recruited in Shanghai, China through posters, phone calls, WeChat, emails and other means between January 2020 and December 2023. A total of 2,208 participants were enrolled, primarily consisting of university students, hospital staff and community residents. The inclusion criteria were non-athletic healthy adults without a history of neurological, endocrine, psychiatric and cardiopulmonary diseases, lower limb injuries or substance abuse. Individuals engaged in specific sports training or regular physical exercise were excluded. All participants were instructed to refrain from vigorous physical activity 48 h before assessment. Informed consent was obtained from all volunteers, and human experiments were conducted following the Helsinki Declaration principles and approved by the Institutional Review Board. The baseline characteristics of the participants are summarised in Table 1.

**TABLE 1:** Participants’ characteristics ( $\bar{x}$ ± s) (n = 2,208).

Sex	Decade of age (years)	n	Age (years)	Height (cm)	Weight (kg)	BMI (kg/m^2^)
Male	20–29	323	25.02 ± 2.8	176.78 ± 6.68	77.83 ± 13.46	24.85 ± 3.68
	30–39	387	34.17 ± 3.03	175.72 ± 5.84	76.73 ± 12.22	24.8 ± 3.37
	40–49	157	42.66 ± 2.55	173.36 ± 5.36	76.46 ± 11.89	25.39 ± 3.36
	50–59	65	53.53 ± 2.63	171.08 ± 5.43	71.39 ± 9.29	24.37 ± 2.83
	60–69	20	67.3 ± 4.79	170.9 ± 3.67	71.3 ± 9.02	24.39 ± 2.83
	All	952	33.85 ± 9.63	175.21 ± 6.25	76.53 ± 12.46	24.88 ± 3.43
Female	20–29	375	23.84 ± 2.93	162.91 ± 5.39	57.17 ± 9.63	21.5 ± 3.19
	30–39	426	33.91 ± 2.78	162.94 ± 5.72	60 ± 10.42	22.58 ± 3.65
	40–49	277	43.05 ± 2.93	161.68 ± 4.83	59.03 ± 7.8	22.6 ± 2.94
	50–59	138	54.24 ± 3.08	160.17 ± 5.96	59.18 ± 8.83	23.01 ± 2.75
	60–69	40	63.97 ± 2.76	158.14 ± 4.6	57.51 ± 6.16	22.98 ± 2.04
	All	1,256	36.59 ± 11.27	162.16 ± 5.53	58.78 ± 9.37	22.34 ± 3.26

Abbreviation: BMI, body mass index.

### Measure of anthropometric characteristics

Prior to the muscle strength assessment, each participant provided their age information and underwent measurements of height and body mass with a medical-grade height and body mass measuring instrument (SH-200G, Shanghe Electronic Technology Co., Ltd., Zhengzhou, China). The participants were instructed to wear only shorts and a vest and to remove shoes, socks and any extra clothing. Thereafter, the participants stood with heels together, hands relaxed and eyes forward. The participants took a deep breath and held it whilst their height and body mass were measured from head to heels. The procedure concluded once the measurements were accurately recorded by the testing personnel. (Gülü and Akalan, 2021). The statistical data detailing anthropometric characteristics for 2,208 participants are categorised by age and gender, as outlined in Table 1.

### Isokinetic testing of knee joint muscle strength

The NX A8-3 isokinetic dynamometer (Yikang, Guangzhou, China) was utilised for random side knee joint strength assessments in all participants (determined through lot selection), intraclass correlation coefficient (ICC) of 0.96 for knee extensors and 0.95 for flexors (Zhang Y. et al., 2023). The participants underwent familiarisation with the testing environment, procedures and contraction modes before formal testing. Before and after the isokinetic assessments, the participants engaged in a 5 min warm-up on a cycle ergometer (NuoCheng, Shanghai, China) at a low load (25 W, 50–60 revolutions per minute) (Zhang Y. et al., 2023). Following isokinetic testing, which can be physically demanding, this “warm-up” acts as a cooldown. The purpose of this routine is to help the body transition back to a resting state, minimizing muscle stiffness and promoting blood circulation. By doing so, it helps clear metabolic byproducts that accumulate during intense exertion, reducing muscle soreness and aiding recovery. This approach not only helps alleviate immediate post-exercise discomfort but also ensures that muscles recover effectively, maintaining their readiness for future activities or training. Incorporating such a routine into post-testing protocols is crucial for maintaining overall muscle health and supporting ongoing athletic performance.

**FIGURE 1:**
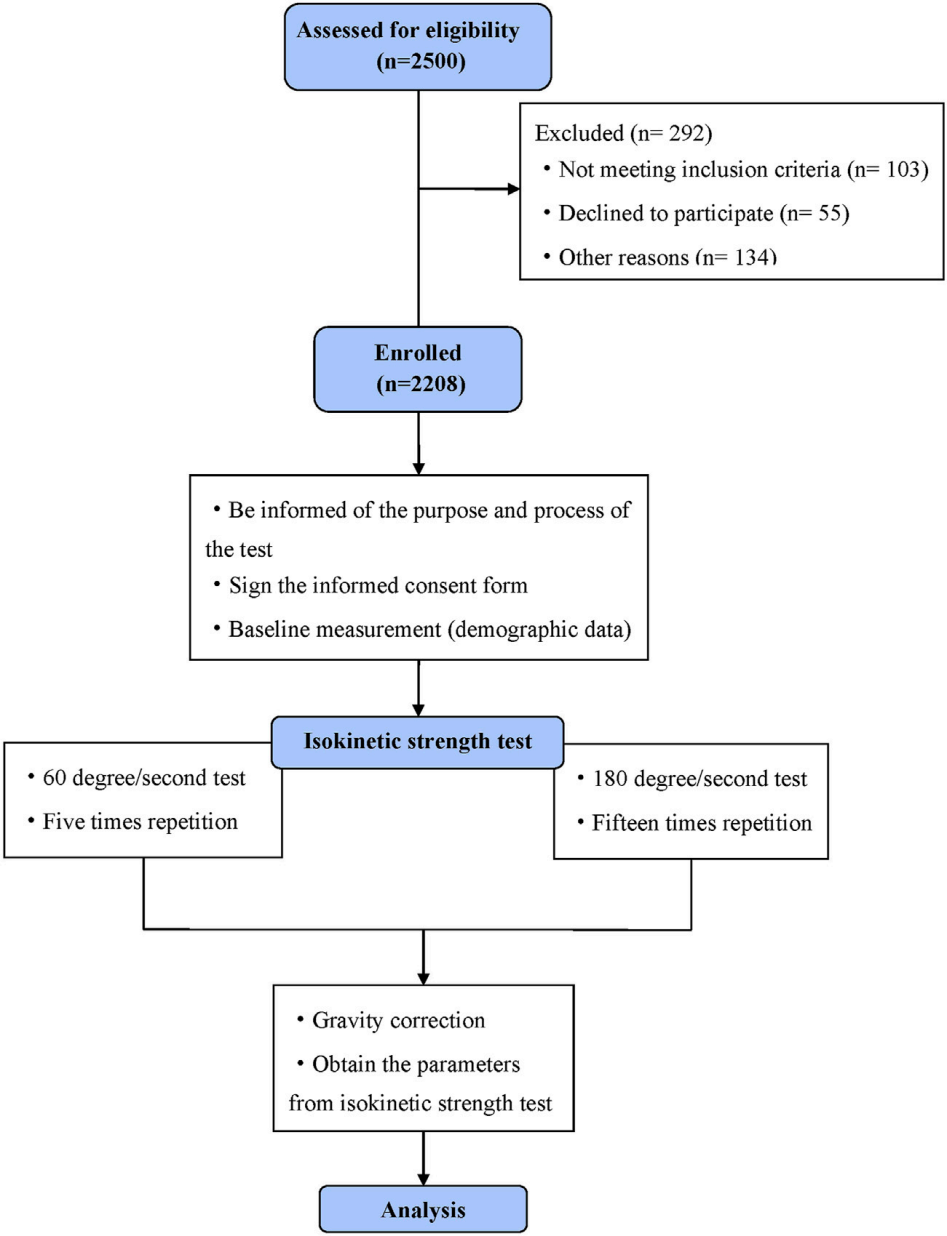
Flow diagram.

As shown in Figure 1, following a detailed elucidation of the testing protocol, the participants assumed a seated position on the dynamometer and meticulously adjusted their posture to ensure a hip joint flexion of 90°, alignment of the lateral femoral condyle with the dynamometer head’s rotation axis and effective fixation of the trunk, pelvis, tested thigh and non-tested lower leg (Kukić et al., 2022). The knee joint range of motion was set from 10° extension to 90° flexion, and gravity correction was applied to mitigate the influence of the lower leg and dynamometer arm weights on the test data.

Isokinetic muscle strength was measured at 60°/s and 180°/s with five repetitions at 60°/s and 15 repetitions at 180°/s, with a 1-min rest interval between trials. The application of 15 repetitions at 180°/s was chosen to ensure accurate measurement by minimizing the effects of variability. For 60°/s, five repetitions were sufficient to reliably capture peak torque, as the lower velocity provides more stable muscle performance (Coban et al., 2021). The recorded values represented the highest peak torque generated by a single muscle contraction throughout the testing process, expressed in absolute values (Nm) and normalised to body weight (Nm/kg). Moreover, the key parameters, such as flexion peak torque/extension peak torque (F/E), peak torque angle and work, were documented. Before the formal testing, four adaptation sessions were conducted at the same angular velocities, involving three submaximal and one maximal isokinetic contraction. The researchers consistently provided verbal encouragement throughout each testing session to ensure that the participants exerted maximal effort.

### Statistical analysis

SPSS 26.0 software was used for statistical analysis. Data were presented as mean ± standard deviation (x̅ ± s). Two-way ANOVA assessed the influence of gender and age on parameters at 60°/s and 180°/s, with Bonferroni *post hoc* analysis and a significance level of p < 0.05. The effect size was reported as ηp^2^, categorized as small (0.01 ≤ ηp^2^ < 0.06), moderate (0.06 ≤ ηp^2^ < 0.14), or large (ηp^2^ ≥ 0.14). Pearson correlation analyzed relationships for normally distributed data, while Spearman correlation was used for non-normal data. Correlation coefficients ranged from −1 to 1, with |*r*| ≥ 0.7 indicating a strong correlation, 0.3 ≤ |*r*| < 0.7 indicating moderate, and |*r*| < 0.3 indicating weak correlations.

Gender, age, height, and weight were independent variables, with isokinetic knee joint torque values including flexion peak torque (FPT) and extension peak torque (EPT) at two angular velocities as dependent variables. We applied linear regression alongside nine machine learning models, including elastic net, K-nearest neighbors, decision tree, random forest, gradient boosting, extreme gradient boosting regressor (XGBR), light gradient boosting machine regressor (LGBMR), support vector machine (SVM), and multilayer perceptron regressor (MLPR). These selected methods aimed to cover a range of model complexities and learning strategies, including linear models with regularization, nonlinear kernel-based models, neural networks, and instance-based learners. All models underwent independent hyperparameter tuning using grid search with five-fold cross-validation to ensure a fair evaluation framework. The machine learning models were selected based on their proven ability to handle large datasets and predict complex outcomes. Metrics such as mean absolute error (MAE), root mean square error (RMSE), mean absolute percentage error (MAPE), and R^2^were calculated for each model, with a higher R^2^value indicating greater explanatory power. The data were divided into five parts, with one part as the validation set and four parts as the training set. Model parameters were optimized through cross-validation to minimize prediction errors. All analyses and developments were performed using Python (version 3.9.7).

## Results

In this study, a total of 2,208 participants are recruited, and the knee joint isokinetic characteristic data are collected at angular velocities of 60°/s and 180°/s. The basic demographics of the participants are presented in Table 1. The selected testing parameters included PT values, peak torque/body weight (PT/BW), peak torque work, F/E and peak torque angle.

**FIGURE 2:**
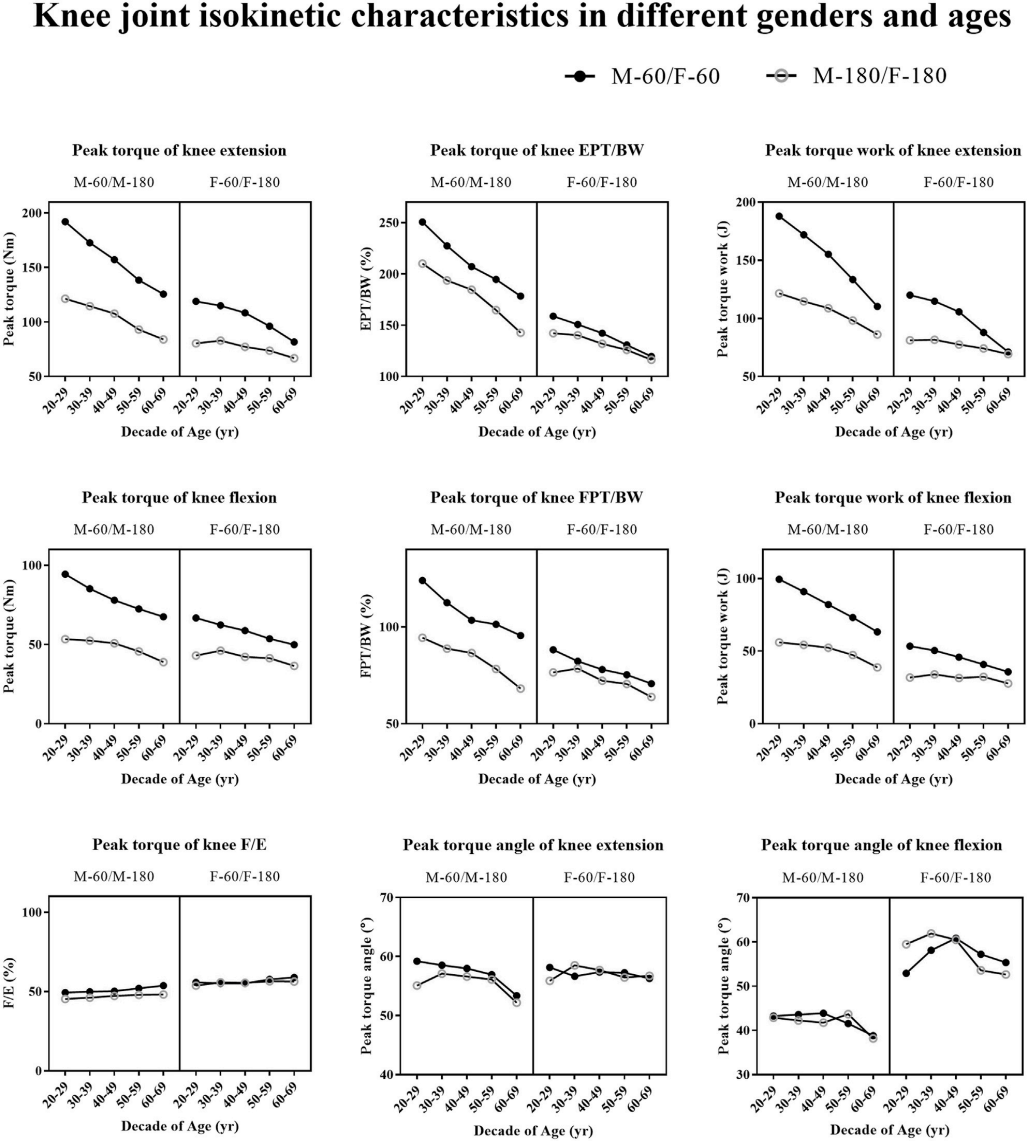
Knee joint isokinetic characteristics in different genders and ages.

**FIGURE 3:**
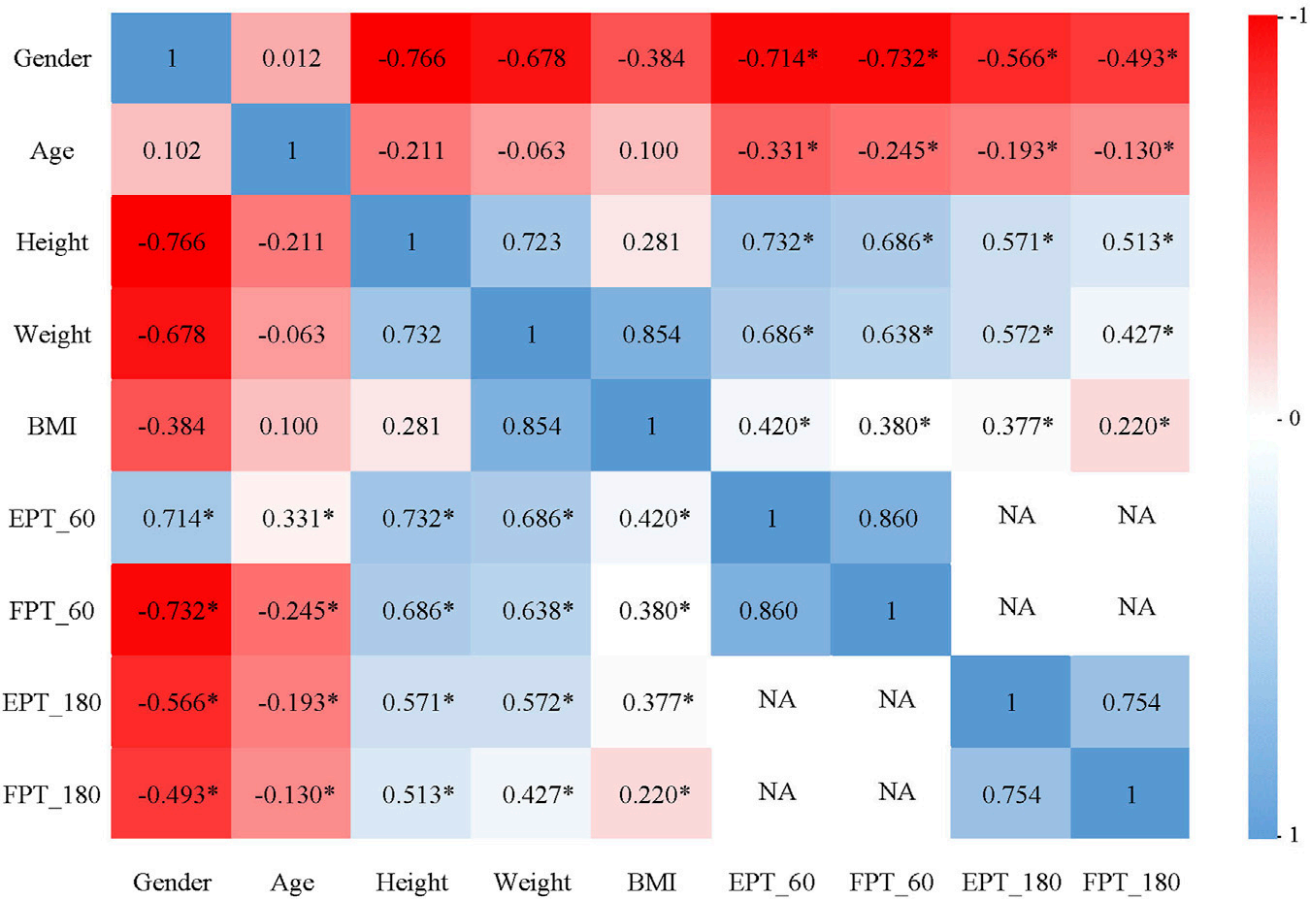
The correlation between isokinetic muscle strength test parameters of knee joint and sex, age, height, weight and BMI at 60°/s and 180°/s.

Overall, all measured characteristics, except for extension peak torque/flexion peak torque, exhibited a decrease with the increase in age. The knee joint isokinetic data obtained at 180°/s were significantly lower than those at 60°/s. Additionally, the males within the same age group demonstrated significantly higher values than females. Moreover, the values for knee joint extension isokinetic are significantly greater than those for flexion isokinetic. The graphical representation in Figure 2 visually depicts these discerned patterns. Figure 3 and Supplementary Tables S1–S4 in Supplement 1 present the specific numerical information.

During the 60°/s isokinetic muscle strength test (Figure 3), a highly significant strong correlation is observed between EPT and gender. EPT exhibited a significantly moderate negative correlation with age, a strongly positive correlation with height and significantly moderate positive correlations with weight and body mass index (BMI). A highly significant strong correlation is observed between FPT and gender. FPT exhibited a significantly weak negative correlation with age and a significantly moderate positive correlations with height, weight and BMI. During the 180°/s isokinetic muscle strength test (Figure 3), a highly significant moderate correlation is observed between EPT and gender. EPT exhibited a significantly weak negative correlation with age and a moderately positive correlation with height, weight and BMI. A highly significant moderate correlation is observed between FPT and gender. FPT exhibited a significantly weak negative correlation with age, a significantly moderate positive correlations with height and weight and significantly weak positive correlations with BMI.

In the machine learning prediction models, the multilayer perceptron regressor exhibited the highest predictive capability. Specifically, the *R* ^2^ values for isokinetic knee flexion and extension movements reached 0.60 at an angular velocity of 60°/s. However, the predictive ability was weaker at an angular velocity of 180°/s, with *R* ^2^ values of 0.37 and 0.25. Table 2 and 3 provides specific details.

**TABLE 2:** Comparison of different prediction model parameters at 60°/s.

Machine Learning Methods	60°/s _extension peak torque				60°/s _flexion peak torque			
	MAE	RMSE	MAPE	*R* ^2^	MAE	RMSE	MAPE	*R* ^2^
LR	22.80	30.57	0.17	0.58	12.08	16.00	0.19	0.59
EN	22.84	30.64	0.17	0.58	12.09	16.00	0.19	0.59
KNN	23.01	30.73	0.17	0.57	12.22	16.10	0.19	0.59
DT	26.81	36.45	0.20	0.40	14.80	20.99	0.23	0.30
RF	23.36	31.13	0.17	0.56	12.51	16.91	0.20	0.54
GB	22.93	30.54	0.17	0.58	12.42	16.35	0.20	0.57
XGBR	26.53	35.72	0.20	0.42	13.87	19.42	0.21	0.40
LGBMR	24.33	32.51	0.18	0.52	13.28	18.12	0.21	0.48
SVM	22.86	32.21	0.16	0.53	12.31	16.84	0.19	0.55
MLPR	22.05	29.63	0.16	0.60	12.05	15.88	0.19	0.60

Abbreviations: LR, linear regression; EN, elastic net; KNN, K-nearest neighbors; DT, decision tree; RF, random forest; GB, gradient boosting; XGBR, extreme gradient boosting regressor; LGBMR, light gradient boosting machine regressor; SVM, support vector machine; MLPR, multilayer perceptron regressor; MAE, mean absolute error; RMSE, root mean squared error; MAPE, mean absolute percentage error; *R* ^2^, *R*-squared.

**TABLE 3:** Comparison of different prediction model parameters at 180°/s.

Machine Learning Methods	180°/s _extension peak torque				180°/s _flexion peak torque			
	MAE	RMSE	MAPE	*R* ^2^	MAE	RMSE	MAPE	*R* ^2^
LR	19.33	25.98	0.25	0.36	14.05	18.63	0.35	0.23
EN	19.36	25.98	0.25	0.36	14.05	18.63	0.35	0.23
KNN	19.60	26.31	0.26	0.34	14.09	18.61	0.34	0.23
DT	22.20	30.92	0.28	0.09	15.08	20.48	0.35	0.07
RF	18.58	26.01	0.25	0.36	13.49	18.74	0.32	0.22
GB	19.28	26.65	0.25	0.33	13.86	18.51	0.34	0.24
XGBR	19.85	27.82	0.26	0.26	13.90	19.04	0.34	0.20
LGBMR	19.42	26.33	0.25	0.34	13.69	18.59	0.34	0.24
SVM	19.30	26.10	0.25	0.35	13.83	18.59	0.33	0.24
MLPR	19.22	25.85	0.25	0.37	13.97	18.47	0.35	0.25

Abbreviations: LR, linear regression; EN, elastic net; KNN, K-Nearest Neighbors; DT, decision tree; RF, random forest; GB, gradient boosting; XGBR, extreme gradient boosting regressor; LGBMR, light gradient boosting machine regressor; SVM, support vector machine; MLPR, multilayer perceptron regressor; MAE, mean absolute error; RMSE, root mean squared error; MAPE, mean absolute percentage error; *R* ^2^, *R*-squared.

## Discussion

In this study, we analysed the normal reference values for isokinetic knee joint flexion and extension muscle strength in non-athletic healthy Chinese adults at speeds of 60°/s and 180°/s, respectively.

We observed a highly significant strong correlation between isokinetic main parameters and gender, alongside a significantly moderate negative correlation with age. In line with previous findings, (Schindler et al., 2023; Benfica et al., 2018; Meldrum et al., 2007), our study found that males exhibited significantly higher isokinetic strength values than females across all age groups, particularly in the knee extensors, with male strength values ranged from 20.47% (60–69 age group) to 38.01% (20–29 age group) higher than females at both 60°/s and 180°/s. In contrast, although knee flexor strength was also higher in males, the difference was relatively larger, ranged from 22.91% (50–59 age group) to 43.42% (20–29 age group). These gender differences may be attributed to greater muscle mass and higher testosterone levels in males, which likely contribute to the enhanced knee extensor strength observed. However, upon further comparison with similar studies, significant discrepancies were noted in the PT values, PT/BW ratio and the F/E ratio at identical angular velocities. These variations may stem from certain factors, such as inclusion of professional athletes (Zvijac et al., 2014; Risberg et al., 2018; Hannon et al., 2022), obese populations (Muollo et al., 2022; Hulens et al., 2002), specific age groups (Holm et al., 2008; Pereira et al., 2019a; Bohannon, 2017), small sample sizes of healthy participants (Harbo et al., 2012; Neder et al., 1999), or studies conducted in previous decades (Dvir, 1996; Perrin et al., 1987; Hulens et al., 2002; Neder et al., 1999; Landers et al., 2001), which may introduce interference in practical applications. Given the scarcity of large-scale studies reporting isokinetic parameters across different genders and age groups amongst non-athletic healthy adults, as well as their interrelationships. Thus, our work aims to provide a comprehensive and targeted reference for clinical research and practice.

The knee joint is crucial for human locomotion, with the quadriceps femoris and hamstring muscles serving as dynamic stabilisers. The ratio of these factors, known as the hamstring/quadriceps ratio (H/Q), is a predictive risk factor for lower limb injuries (Dallinga et al., 2012) and one of the criteria for safe return to sports activities (Erickson and Sherry, 2017). Studies have reported H/Q ratios ranging from 0.50 to 0.80 at 60°/s, close to 0.60, increasing with angular velocity (Evangelidis et al., 2015; Ghena et al., 1991; Rivera-Brown et al., 2022). ratio below 0.60 indicates potential muscular imbalance and injury risk (Coombs and Garbutt, 2002; Andrade Mdos et al., 2012). However, our study found H/Q ratios ranged from 0.49 to 0.53 for males and 0.45 to 0.48 for females at 60°/s, remaining below 0.60 even at 180°/s. Muollo et al. (2022) study reported ratios below 0.50, although their subjects were elderly obese and non-obese individuals. This deviation may be due to previous studies primarily involving professional athletes with superior fitness levels (Zvijac et al., 2014; Risberg et al., 2018; Hannon et al., 2022; Wilkosz et al., 2021; Chen et al., 2023), who generally possess better physical fitness than non-athletic healthy individuals. In sports, such as soccer, running athletes have highly developed lower limb muscle strength. Although an H/Q ratio of 0.50 may be common in the general population for daily activities, ratios exceeding 0.60 are typical training goals for athletes. Additionally, variations in body composition amongst different races and populations may influence muscle strength testing, suggesting potential strength differences amongst ethnicities (Baker et al., 2013; Zengin et al., 2015). These study findings may be more applicable to populations in China, East Asia and Southeast Asia for reference due to similar genetic backgrounds, lifestyles, and environmental factors.

In traditional isokinetic analyses, the consideration of knee joint flexion or extension angles corresponding to PT measurements is often neglected. The PT angles during knee flexion and extension movements vary. This discrepancy has been noted by Baumgart et al. (2018) Such findings suggest that muscle strength contributing to the H/Q ratio may not occur at the same angle, potentially inadequately reflecting joint stability. Aagaard et al. (1998); Aagaard et al. (1995) proposed using the “functional ratio” or “dynamic control ratio” to assess the muscle balance of knee flexors and extensors. This ratio, calculated as “eccentric hamstring torque/concentric quadriceps torque,” aims to better prevent hamstring strains and anterior cruciate ligament injuries during movement. Given that hamstring strains typically occur during the terminal phase of knee extension when the hamstrings exert maximal eccentric contraction, the “eccentric hamstring peak torque/concentric quadriceps peak torque” ratio holds more scientific diagnostic value and significance than the “concentric hamstring peak torque/concentric quadriceps peak torque” ratio (Cao, 2017).

At 60°/s speed, we observed a significant moderate positive correlation between body weight and peak torque, emphasising the need to account for the body weight’s influence on muscle strength. Accordingly, we calculated the relative peak torque by dividing peak torque (Nm) by body weight (kg) and multiplying by 100%. This normalization method (PT/BW) is widely used due to its simplicity and ease of application in both clinical and large-scale field studies (Risberg et al., 2018). While alternative normalization techniques such as allometric scaling or limb muscle cross-sectional area (CSA) may offer more individualized control for body composition (Mathur et al., 2010; Bazett-Jones et al., 2011), they often require complex measurements or imaging, which are not always practical in population-based research settings. Jaric et al. (2002) recommended dividing torque values by body mass, whilst force values should be divided by two-thirds of the body mass to facilitate accurate inter-participant comparisons. Abdalla et al. (2020) suggested that when determining muscle wasting, knee extension strength should be divided by 96% of body weight for elderly men and 70% for elderly women. In children, 140% of their body mass should be used in the division process (Wren and Engsberg, 2007). Although the idea of whether normalising peak torque by body weight is always optimal remains unclear, several studies have indicated that the relative peak torque is a valuable reference index. Future researchers should distinguish between absolute and relative peak torque when citing or referencing these findings (Šarabon et al., 2021). Moreover, body weight-based normalization may introduce systematic bias in individuals with disproportionate fat-to-muscle mass ratios (Tomlinson et al., 2016). Future studies aiming for greater physiological precision may consider integrating allometric scaling or including direct muscle mass estimates (e.g., *via* DXA or MRI) to enhance the comparability and accuracy of inter-individual torque assessments. Furthermore, we found a strong positive correlation between height and PT values, consistent with previous research, further affirming height as a predictor of knee joint isokinetic peak torque (Harbo et al., 2012; Neder et al., 1999; Gross et al., 1989). Leg length determines the lever arm during knee joint motion, thus influencing force output. Accordingly, future studies predicting knee joint isokinetic reference values or equations may consider leg length as a separate factor.

Although our primary analysis focused on the magnitude of peak torque, we recognize that the angle at which peak torque occurs can vary substantially between individuals. This variability may have important biomechanical and clinical implications. In our study, we recorded and summarized the corresponding peak torque angles across different age groups and angular velocities (see Supplementary Tables S1–S2), which provide preliminary evidence of such inter-individual variation. These angle-specific data warrant further investigation, and future studies will aim to analyze the full torque–angle profiles to better understand their potential role in functional assessment and clinical decision-making. Additionally, we assessed muscle work, which exhibited similar variations to PT values or PT/BW ratio. This supplementary data may offer insights more closely aligned with functional performance. We look forward to the broader application and further investigation of this aspect.

To our knowledge, this study is the first to apply machine learning models to predict the isokinetic muscle strength values (FPT and EPT) of the knee joint. Our findings suggest that machine learning models exhibit stronger predictive capabilities compared with linear regression equations, with MLPR demonstrating the highest predictive performance amongst various models. The diverse model selection enabled us to evaluate the impact of various learning strategies on prediction accuracy. (Chen et al., 2024). MLPR outperformed the other models likely due to its ability to capture complex nonlinear relationships in the data, while other models such as LR, EN, KNN performed well in handling regularized and linear patterns (Abbas et al., 2022). Hyperparameter tuning through grid search and cross-validation ensured that each model was optimized fairly, contributing to the reliability of the performance comparison. Despite our relatively large sample size, the predictive ability of our machine learning models, particularly at 180°/s, was modest (*R* ^2^ = 0.25–0.37), compared to the higher accuracy observed at lower velocities (e.g., *R* ^2^ = 0.60 at 60°/s). This discrepancy may be attributed to the nature of high-speed contractions, which are more sensitive to neuromuscular coordination, reaction time, and tendon elasticity—factors not captured in our input variables. Furthermore, our models did not include several important physiological predictors known to influence muscle strength, such as muscle volume, fiber type composition, fascicle length, tendon stiffness, and neural activation strategies. (O’Brien et al., 2010; Jiang and Wang, 2003).

These omissions likely contributed to the limited performance at higher angular velocities, where explosive strength and neuromechanical factors play a larger role. While our models were based primarily on demographic and anthropometric variables for simplicity and clinical feasibility, future work should explore the incorporation of advanced physiological parameters—potentially obtained *via* imaging (e.g., MRI for muscle volume) or EMG—to improve model accuracy, especially for applications in high-performance or rehabilitation settings (O’Brien et al., 2010; Zhang J. et al., 2023). Nevertheless, the provided models and reference values still serve as useful screening tools in general populations for initial assessment and risk stratification.

This study is strengthened by its large sample size of 2,208 participants, which enhances the generalizability of the normative values. The innovative application of machine learning for prediction models offers a robust approach to understanding knee strength in a non-athletic population. However, as a single-center study, the findings may not fully represent broader populations. Additionally, the lack of isometric and eccentric contraction tests limits the comprehensiveness of our strength assessment. Isometric strength is often used as a reliable measure of baseline muscle capacity, while eccentric strength plays a crucial role in deceleration movements and injury prevention, particularly in tasks involving sudden changes in direction or landing (Swales et al., 2023; Hu et al., 2023). The absence of these contraction types may reduce the applicability of our findings in certain clinical and athletic contexts. Future studies should incorporate isometric and eccentric modalities to capture a more holistic profile of neuromuscular function and provide deeper insight into injury risk and functional capacity. Despite these limitations, the normative values obtained in this study can assist clinicians in assessing knee health and personalizing rehabilitation strategies based on individual muscle strength profiles. For rehabilitation professionals, understanding these reference values is crucial for designing effective treatment plans. Future research could benefit from incorporating variables such as muscle volume or fiber type to provide a more comprehensive model of knee strength prediction. Additionally, although locomotion functions (e.g., gait speed, stride length) were not assessed in this study, future work will examine the relationship between isokinetic strength and functional mobility outcomes to enhance clinical applicability. Multi-center studies and the inclusion of underrepresented groups would further enhance the generalizability and applicability of the findings.

## Conclusion

This study provides comprehensive normative isokinetic muscle strength reference values and prediction models for the knee joint in non-athletic healthy Chinese adults. The muscle strength characteristics of the knee extensor and flexor groups at various angular velocities exhibit significant age and gender differences, with height, weight and body mass index also exerting some influence on these characteristics. These findings offer valuable data for clinical assessments and personalized rehabilitation strategies to improve knee joint health and overall quality of life.

## Data Availability

The raw data supporting the conclusions of this article will be made available by the authors, without undue reservation.

## Abbreviations

PT peak torque; ICC intraclass correlation coefficient; F/E flexion peak torque/extension peak torque; FPT flexion peak torque; EPT extension peak torque; XGBR extreme gradient boosting regressor; LGBMR light gradient boosting machine regressor; MLPR multilayer perceptron regressor; SVM support vector machine; MAE mean absolute error; RMSE root mean square error; MAPE mean absolute percentage error.; PT/BW peak torque/body weight; BMI body mass index; H/Q hamstring/quadriceps; CSA cross-sectional area; MRI magnetic resonance imaging; DXA dual-energy x-ray absorptiometry; EMG electromyography.
