# Supplementary material for: Normative isokinetic knee strength values and prediction models in non-athletic Chinese adults
Source: Front Bioeng Biotechnol. 2025 May 21;13:1573267. doi: 10.3389/fbioe.2025.1573267 (PMC12133959; doi:10.3389/fbioe.2025.1573267)
Supplement: Supplementary file 1 [file Table1.docx]

eTable 1. Isokinetic muscle strength test parameters of knee joint at 60°/s

eTable 2. Isokinetic muscle strength test parameters of knee joint at 180°/s

eTable 3. The correlation between isokinetic muscle strength test parameters of knee joint and sex, age, height, weight and BMI at 60°/s.

eTable 4. The correlation between isokinetic muscle strength test parameters of knee joint and sex, age, height, weight and BMI at 180°/s.

eTable 1 Isokinetic muscle strength test parameters of knee joint at 60°/s

|  | Groups | EPT (Nm) | FPT (Nm) | F/E (%) | EPT/BW (%) | FPT/BW (%) | EPT-a (°) | FPT-a (°) | EPT-w (J) | FPT-w (J) |
| --- | --- | --- | --- | --- | --- | --- | --- | --- | --- | --- |
| Male | 20-29 | 192.02±38.83 | 94.4±23.9 | 49.35±9.08 | 250.61±50.64 | 124±34.86 | 59.2±8.21 | 43.25±13.14 | 187.95±41.1 | 99.49±28.35 |
|  | 30-39 | 172.73±36.81 | 85.17±19.78 | 49.92±9.33 | 227.37±45.72 | 112.48±26.26 | 58.55±8.48 | 43.59±12.83 | 171.94±38.55 | 90.91±24.02 |
|  | 40-49 | 157.27±34.98 | 78±16.69 | 50.31±8.77 | 207.17±40.59 | 103.46±23.04 | 57.98±8.9 | 43.9±12.24 | 155.22±34.11 | 82.05±19.3 |
|  | 50-59 | 138.47±28.34 | 72.45±23.05 | 52.06±12.11 | 194.65±35.48 | 101.31±28.5 | 56.94±7.82 | 41.56±12.63 | 133.49±34.18 | 73.16±28.44 |
|  | 60-69 | 125.67±20.86 | 67.54±17.84 | 53.76±10.59 | 178.42±33.27 | 95.54±25.18 | 53.37±11.94 | 38.83±15.4 | 110.3±35.11 | 63.25±25.96 |
| Female | 20-29 | 119.03±25.95 | 53.41±12.41 | 45.32±7.4 | 210.13±39.45 | 94.47±20.18 | 55.09±7.46 | 42.87±13.81 | 121.61±27.48 | 56.08±14 |
|  | 30-39 | 115.06±25.22 | 52.52±12.64 | 46.11±7.97 | 193.78±37.87 | 88.84±21.21 | 57.1±7.66 | 42.26±13.01 | 114.72±27.87 | 54.31±15.54 |
|  | 40-49 | 108.49±21.24 | 50.81±12.02 | 47.26±9.06 | 184.63±31.98 | 86.6±19.39 | 56.6±7.77 | 41.75±11.42 | 108.87±23.3 | 52.36±14.14 |
|  | 50-59 | 96.26±20.37 | 45.67±11.03 | 47.93±9.09 | 164.84±36.02 | 78.31±20.17 | 56.12±8.16 | 43.69±15.96 | 98.24±20.67 | 47.4±12.75 |
|  | 60-69 | 81.82±14.15 | 38.93±8.34 | 48.15±9.06 | 142.71±23.81 | 68.18±15.36 | 52.19±9.78 | 38.19±13.05 | 86.21±18.45 | 38.87±11.09 |
| *P*-value/η *p*² | gender | < 0.001*/0.248 | < 0.001*/0.269 | < 0.001*/0.023 | < 0.001*/0.063 | < 0.001*/0.092 | < 0.001*/0.014 | 0.611/0.000 | < 0.001*/0.178 | < 0.001*/0.212 |
|  | age | < 0.001*/0.156 | < 0.001*/0.086 | 0.001*/0.010 | < 0.001*/0.159 | < 0.001*/0.075 | 0.185/0.003 | 0.216/0.003 | < 0.001*/0.151 | < 0.001*/0.088 |
|  | gender×age | < 0.001*/0.030 | < 0.001*/0.026 | 0.853/0.001 | 0.026*/0.006 | 0.005*/0.008 | 0.003*/0.009 | 0.494/0.002 | < 0.001*/0.028 | < 0.001*/0.023 |

EPT, extension peak torque; FPT, flexion peak torque; F/E, flexion peak torque/ extension peak torque; EPT/BW, extension peak torque/body weight; FPT/BW, flexion peak torque/body weight; EPT-a, extension peak torque angle, FPT-a, flexion peak torque angle; EPT-w, extension peak torque work; FPT-w, flexion peak torque work. η *p²*, effect size; *Significant difference between groups, *p* < 0.05.

eTable 2 Isokinetic muscle strength test parameters of knee joint at 180°/s

|  | Groups | EPT (Nm) | FPT (Nm) | F/E (%) | EPT/BW (%) | FPT/BW (%) | EPT-a (°) | FPT-a (°) | EPT-w (J) | FPT-w (J) |
| --- | --- | --- | --- | --- | --- | --- | --- | --- | --- | --- |
| Male | 20-29 | 121.45±34.97 | 66.74±23 | 55.8±13.99 | 158.83±45.77 | 88.24±33.08 | 58.16±8.05 | 52.9±30.02 | 120.1±38.29 | 53.4±22.91 |
|  | 30-39 | 114.58±28.57 | 62.36±18.32 | 55.16±13.38 | 150.78±35.92 | 82.31±24.13 | 56.67±9.03 | 58.11±29.27 | 114.76±30.99 | 50.47±18.69 |
|  | 40-49 | 107.75±25.78 | 58.82±16.54 | 55.15±11.93 | 142.34±32.82 | 78.04±22.52 | 57.35±8.16 | 60.85±29.26 | 105.63±28.66 | 45.9±17.94 |
|  | 50-59 | 93.13±22.01 | 53.68±16.52 | 57.7±11.78 | 130.8±26.76 | 75.35±21.09 | 57.26±12.31 | 57.22±29.91 | 87.94±28.81 | 40.86±20.55 |
|  | 60-69 | 84.01±12.54 | 49.93±14.6 | 58.9±11.85 | 119.68±23.4 | 70.7±21.03 | 53.3±12.58 | 55.34±29.03 | 71.17±24.22 | 35.77±18.61 |
| Female | 20-29 | 80.47±17.31 | 43.03±13.59 | 53.8±13.58 | 142.33±28.31 | 76.48±23.51 | 55.89±9.85 | 59.47±29.77 | 81.24±19.87 | 31.83±11.82 |
|  | 30-39 | 83.01±20.34 | 46.11±15.36 | 55.81±13.89 | 140.3±34.48 | 78.55±28.68 | 58.51±9.78 | 61.92±29.67 | 81.74±22.51 | 34.05±14.47 |
|  | 40-49 | 77.3±20.56 | 42.22±13.6 | 55.47±14.39 | 131.98±35.86 | 72.26±23.77 | 57.7±9.71 | 60.47±29.01 | 77.58±22.1 | 31.52±12.41 |
|  | 50-59 | 73.77±22.28 | 41.38±15.44 | 56.48±13.88 | 126.06±38.21 | 70.62±26.88 | 56.46±13.92 | 53.58±29.32 | 74.24±22.88 | 32.37±14.21 |
|  | 60-69 | 66.81±25.1 | 36.57±13.26 | 56.34±15.57 | 116.42±41.58 | 63.85±22.13 | 56.74±12.18 | 52.65±30.32 | 69.31±30.49 | 27.77±17.13 |
| *P*-value/η *p*² | gender | < 0.001*/0.117 | < 0.001*/0.091 | 0.322/0.001 | < 0.001*/0.007 | < 0.001*/0.007 | 0.898/0.000 | 0.950/0.000 | < 0.001*/0.069 | < 0.001*/0.066 |
|  | age | < 0.001*/0.058 | < 0.001*/0.027 | 0.290/0.003 | < 0.001*/0.046 | < 0.001*/0.020 | 0.757/0.001 | 0.042*/0.005 | < 0.001*/0.061 | < 0.001*/0.022 |
|  | gender×age | < 0.001*/0.018 | < 0.001*/0.013 | 0.456/0.002 | 0.279/0.003 | 0.103/0.004 | 0.007/0.008 | 0.161/0.004 | < 0.001*/0.023 | < 0.001*/0.014 |

EPT, extension peak torque; FPT, flexion peak torque; F/E, flexion peak torque/ extension peak torque; EPT/BW, extension peak torque/body weight; FPT/BW, flexion peak torque/body weight; EPT-a, extension peak torque angle, FPT-a, flexion peak torque angle; EPT-w, extension peak torque work; FPT-w, flexion peak torque work. η *p²*, effect size; *Significant difference between groups, *p* < 0.05.

eTable 3 The correlation between isokinetic muscle strength test parameters of knee joint and sex, age, height, weight and BMI at 60°/s.

|  |  | EPT (Nm) | FPT (Nm) | F/E (%) | EPT/BW (%) | FPT/BW (%) | EPT-a (°) | FPT-a (°) | EPT-w (J) | FPT-w (J) |
| --- | --- | --- | --- | --- | --- | --- | --- | --- | --- | --- |
| r | Gender | -0.714 | -0.732 | -0.206 | -0.375 | -0.452 | -0.157 | -0.028 | -0.660 | -0.687 |
|  | Age | -0.331 | -0.245 | 0.071 | -0.403 | -0.268 | -0.033 | -0.026 | -0.330 | -0.250 |
|  | Height | 0.723 | 0.686 | 0.101 | 0.354 | 0.365 | 0.103 | 0.090 | 0.681 | 0.653 |
|  | Weight | 0.686 | 0.638 | 0.059 | 0.085 | 0.118 | 0.093 | 0.012 | 0.665 | 0.603 |
|  | BMI | 0.420 | 0.380 | 0.003 | -0.149 | -0.113 | 0.052 | -0.036 | 0.422 | 0.356 |
| *p* | Gender | < 0.001* | < 0.001* | < 0.001* | < 0.001* | < 0.001* | < 0.001* | 0.235 | < 0.001* | < 0.001* |
|  | Age | < 0.001* | < 0.001* | 0.002* | < 0.001* | < 0.001* | 0.159 | 0.260 | < 0.001* | < 0.001* |
|  | Height | < 0.001* | < 0.001* | < 0.001* | < 0.001* | < 0.001* | < 0.001* | < 0.001* | < 0.001* | < 0.001* |
|  | Weight | < 0.001* | < 0.001* | 0.011* | < 0.001* | < 0.001* | < 0.001* | 0.610 | < 0.001* | < 0.001* |
|  | BMI | < 0.001* | < 0.001* | 0.887 | < 0.001* | < 0.001* | 0.025* | 0.117 | < 0.001* | < 0.001* |

*r,* spearman correlation coefficient, EPT, extension peak torque; FPT, flexion peak torque; F/E, flexion peak torque/ extension peak torque; EPT/BW, extension peak torque/body weight; FPT/BW, flexion peak torque/body weight; EPT-a, extension peak torque angle, FPT-a, flexion peak torque angle; EPT-w, extension peak torque work; FPT-w, flexion peak torque work. *Significant difference between groups, *p* < 0.05.

eTable 4 The correlation between isokinetic muscle strength test parameters of knee joint and sex, age, height, weight and BMI at 180°/s.

|  |  | EPT (Nm) | FPT (Nm) | F/E (%) | EPT/BW (%) | FPT/BW (%) | EPT-a (°) | FPT-a (°) | EPT-w (J) | FPT-w (J) |
| --- | --- | --- | --- | --- | --- | --- | --- | --- | --- | --- |
| r | Gender | -0.566 | -0.493 | -0.024 | -0.218 | -0.170 | 0.011 | 0.055 | -0.500 | -0.459 |
|  | Age | -0.193 | -0.130 | 0.046 | -0.250 | -0.145 | 0.025 | 0.034 | -0.191 | -0.120 |
|  | Height | 0.571 | 0.513 | 0.043 | 0.187 | 0.166 | 0.005 | 0.004 | 0.500 | 0.414 |
|  | Weight | 0.572 | 0.427 | -0.089 | -0.028 | -0.087 | -0.065 | -0.107 | 0.519 | 0.398 |
|  | BMI | 0.377 | 0.220 | -0.154 | -0.177 | -0.243 | -0.087 | -0.149 | 0.354 | 0.249 |
| *p* | Gender | < 0.001* | < 0.001* | 0.311 | < 0.001* | < 0.001* | 0.628 | 0.018* | < 0.001* | < 0.001* |
|  | Age | < 0.001* | < 0.001* | 0.050 | < 0.001* | < 0.001* | 0.283 | 0.149 | < 0.001* | < 0.001* |
|  | Height | < 0.001* | < 0.001* | 0.063 | < 0.001* | < 0.001* | 0.827 | 0.868 | < 0.001* | < 0.001* |
|  | Weight | < 0.001* | < 0.001* | < 0.001* | 0.228 | < 0.001* | 0.005* | < 0.001* | < 0.001* | < 0.001* |
|  | BMI | < 0.001* | < 0.001* | < 0.001* | < 0.001* | < 0.001* | < 0.001* | < 0.001* | < 0.001* | < 0.001* |

*r,* spearman correlation coefficient, EPT, extension peak torque; FPT, flexion peak torque; F/E, flexion peak torque/ extension peak torque; EPT/BW, extension peak torque/body weight; FPT/BW, flexion peak torque/body weight; EPT-a, extension peak torque angle, FPT-a, flexion peak torque angle; EPT-w, extension peak torque work; FPT-w, flexion peak torque work. *Significant difference between groups, *p* < 0.05.
